# Hunk/Mak-v is a negative regulator of intestinal cell proliferation

**DOI:** 10.1186/s12885-015-1087-2

**Published:** 2015-03-08

**Authors:** Karen R Reed, Igor V Korobko, Natalia Ninkina, Elena V Korobko, Ben R Hopkins, James L Platt, Vladimir Buchman, Alan R Clarke

**Affiliations:** 1 University of Cardiff, European Cancer Stem Cell Research Institute, School of Biosciences, Cardiff, CF10 3AX UK; 2 School of Biosciences, University of Cardiff, Cardiff, CF10 3AX UK; 3 Russian Academy of Sciences, Institute of Gene Biology, 34/5 Vavilov street, Moscow, 119334 Russia; 4 Institute of General Pathology and Pathophysiology of Russian Academy of Medical Science, 8 Baltijskaya str, Moscow, 125315 Russia

**Keywords:** Hunk, Mak-V, Intestine, Wnt signalling, Cancer, Apc^Min^ microarray

## Abstract

**Background:**

Conditional deletion of the tumour suppressor gene *Apc* within the murine intestine results in acute Wnt signalling activation. The associated over-expression of a myriad of Wnt signalling target genes yields phenotypic alterations that encompass many of the hallmarks of neoplasia. Previous transcriptomic analysis aimed at identifying genes that potentially play an important role in this process, inferred the Hormonally upregulated Neu-associated kinase (*HUNK/Mak-v/Bstk1*) gene as a possible candidate. Hunk is a SNF1 (sucrose non fermenting 1)-related serine/threonine kinase with a proposed association with many different tumour types, including colorectal cancer.

**Methods:**

Here we describe the generation of a novel Hunk kinase deficient mouse which has been used to investigate the involvement of Hunk-kinase activity in intestinal homeostasis and tumourigenesis.

**Results:**

We show that in the morphologically normal intestine, Hunk-kinase negatively regulates epithelial cell proliferation. However, the increase in cell proliferation observed in the Hunk kinase deficient intestine is counteracted by increased cell migration, thereby maintaining intestinal homeostasis. Using qRT-PCR, we further demonstrate that *Hunk* is significantly over-expressed in *Apc* deficient / Wnt-signalling activated intestinal tissue. Using the classical intestinal tumourigenesis *Apc*^*Min*^ mouse model we show that loss of Hunk-kinase activity significantly reduced tumour initiation rates in the small intestine. However, an accompanying increase in the size of the tumours counteracts the impact this has on overall tumour burden or subsequently survival.

**Conclusions:**

In the intestinal setting we demonstrate that *Hunk* has a role in normal intestinal proliferation and homeostasis and, although it does not alter overall survival rates, activity of this kinase does impact on tumour initiation rates during the early stages in tumourigenesis in the small intestine.

**Electronic supplementary material:**

The online version of this article (doi:10.1186/s12885-015-1087-2) contains supplementary material, which is available to authorized users.

## Background

Activation of the Wnt signalling pathway is a recognised early event in many intestinal cancers. Mouse models of intestinal neoplasia have proven to be invaluable in increasing our knowledge and understanding relating to the contribution of individual genes in this process. We have previously used Cre-Lox technology to conditionally delete the *Apc* gene in the mouse intestine and characterised the phenotypic and transcriptional changes that occur following the acute activation of Wnt signalling in this tissue [1]. Our microarray analysis demonstrated transcriptional activation of the Hormonally upregulated Neu-associated kinase (*HUNK/Mak-v/Bstk1*) gene immediately following *Apc* loss, indicating that *Hunk* is potentially a Wnt signalling target gene which could play a role during the initial stages of intestinal neoplasia. Hunk is a SNF1(sucrose non fermenting 1)-related serine/ threonine kinase that was originally cloned by Korobko *et al.* [2,3] and Gardner *et al.* [4] but its function still remains largely unknown. A variety of binding partners for Hunk have been identified including Nedd4 E3 ubiquitin ligase [5], Synaptopodin [6], Rabaptin-5 [7] and cofilin-1 [8], although the molecular mechanisms of Hunk action remain unclear. *Hunk* has been shown to be expressed in a variety of tissues but is most notably associated with pregnancy-induced alterations in the mammary gland and high levels of expression within the brain [4,9]. Two independent studies have shown that Hunk is able to negatively regulate proliferation in normal epithelial cells. Gain-of-function and loss-of-function studies within mouse distal convoluted tubule (mDCT) cells, demonstrated that Hunk negatively regulates ANG II-induced c-fos gene expression and mDCT proliferation [10]. Furthermore, MMTV-driven Hunk over-expression within mammary epithelium, inhibits proliferation of alveolar epithelial cells during mid-pregnancy [9]. However, within the cancer setting, both pro- and antitumourigenic properties for Hunk have been described. Overexpression of *Hunk* has been shown in a number of different cancers, and it is thought to be associated with the more aggressive subset of carcinomas [11,12], probably due to its ability to support cell viability and survival [3,13,14]. Using transgenic mouse models, Yeh *et al.* [13] have shown that *Hunk* plays a role in tumour initiation and is required to facilitate HER2/neu-induced mammary tumourigensis. Contrary to this, Wertheim *et al.* [12] demonstrated that *Hunk* was dispensable for tumour initiation in a MMTV-cMyc driven model of mammary tumourigenesis, but was essential for tumour metastasis, and therefore impacted on overall survival in this mouse tumour model. Both of these studies suggest Hunk functions in a pro-tumourigenic manner. Conversely, in a xenograft model of mammary tumourigenesis using a basal breast cancer cell line in which *Hunk* was over expressed, Quintela-Fandino *et al.* [8] demonstrate that Hunk overexpression suppresses metastasis, suggesting a tumour suppressor role for Hunk. However, the differences in the experimental setup of these studies make it difficult to draw any firm conclusions as to the role of Hunk in tumourigenesis. Although over-expression of *Hunk* has been shown to be associated with advanced and aggressive forms of carcinoma [12], no one to date has studied the importance of *Hunk* in intestinal tumourigenesis. Indeed, analysis of the Oncomine database confirmed the association of *Hunk* expression and intestinal cancer. For breast cancer, the cancer conventionally associated with Hunk, 1 out of 27 analyses (3.7%) within the Oncomine database demonstrate greater than 1.5 fold over-expression of *Hunk* (p < 0.01). However, using the same cut-off criteria, 5 out of 24 analyses (28.8%) associated with colorectal cancer. This clearly indicates that overexpression of *Hunk* is potentially important in intestinal cancer and warrants further investigation.

Loss of the tumour suppressor protein adenomatosis polyposis coli (APC) and activation of the Wnt signalling pathway is recognised as an early key event in the majority of intestinal neoplasia. Work within Xenopus embryos has demonstrated that Hunk has the ability to modulate Wnt signalling, which is presumed to be via Hunk directed phosphorylation of Disheveled [15]. Here we describe the generation of a novel Hunk-kinase deficient mouse, and the subsequent investigation of the contribution of Hunk-kinase in normal intestinal homeostasis and tumourigenesis using this novel knock-out.

## Methods

### Targeting construct

**Figure 1:**
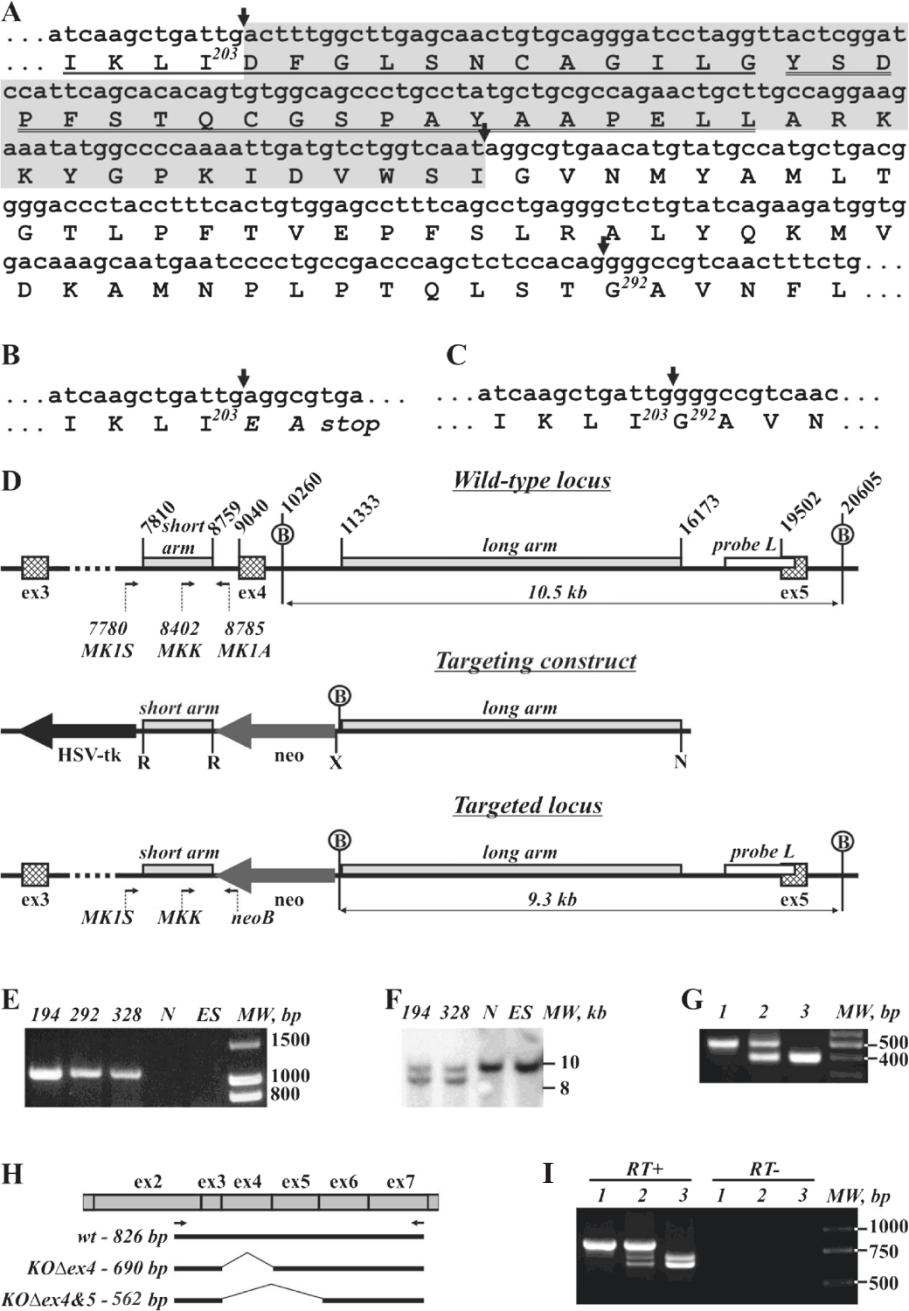
**Strategy for targeting the*Hunk*allele. A)** Nucleotide sequence of mouse *Hunk* cDNA corresponding to targeted exon 4 (highlighted). Arrows mark exon/intron junctions. Subdomains VII (single-underline) and VIII (double-underline) of the catalytic domain are indicated. **B, C** Nucleotide sequences of *Hunk* cDNA with spliced out exon 4 **(B)** or exons 4 and 5 **(C)**. Note, a frame shift and truncation protein truncation occurs following splicing between exons 3 and 5. However, splicing between exons 3 and 6 (which doesn’t induce a frame shift), produces a protein lacking subdomains important for kinase activity. **D** Schematic of the targeting strategy. Hatched boxes represent exons. EcoRI (*R*), XhoI (*X*), NotI (*N*) sites (for cloning “short” and “long arms” into pPNT vector) and BamHI **(B)** sites (for “long arm” recombination analysis) are shown. Arrows represent primers (used for ES clone analysis and genotyping of mice). Nucleotide positions are shown according to mouse chromosome 16 sequence GeneBank Acc. No. NT 039625 2. Neomycin phopshotransferase (dark grey arrow) and HSV thymidine kinase (black arrow) expression cassettes, are also shown. **E** PCR analysis of ES clones (primers MK1S and neoB) demonstrating homologous recombination of the “short arm” in clones 194, 292 and 328 but not in negative clone N or parental E14Tg2a ES cells (*ES*). **F** Southern blot analysis using P32-labeled probe “L” (white box in panel **D**) and BamHI-digested genomic DNA from clones 194 and 328, negative clone N and parental E14Tg2a ES cells (*ES*). **G** PCR genotyping of *Hunk*−/−(lane*1*), *Hunk*+/−(lane*2*) and Hunk+/+(lane*3*) mice (primers MK1A, MKK and neoB). **H** Scheme of exons 2 through 7 of *Hunk* cDNA, demonstrating amplification products from wild type allele (*wt*), targeted allele missing exon 4 (*KODex4*) and with additionally spliced out exon 5 (*KODex4&5*). Arrows represent RT-PCR Primers. **I** RT-PCR amplification from cerebellum RNA of *Hunk*+/+(lane*1*), *Hunk*+/−(lane*2*) and *Hunk*−/−(lane*3*) mice. *MW* – DNA molecular weight markers.

A plasmid construct for targeting exon 4 of the mouse *Hunk* gene (Figure 1D) was generated using pPNT vector [16] as a backbone with 0.95 kb “short arm” cloned into EcoRI site and 4.84 kb “long arm” cloned between XhoI and NotI sites. “Short” and “long arms” were obtained by PCR amplification of E14Tg2a embryonic stem (ES) cell genomic DNA with high-fidelity Platinum Pfx or AccuPrime polymerases (Invitrogen) and specific primers designed on the basis of C57Bl/6 J genome sequence (GeneBank Acc.No. NT 039625 2).

### Targeting *Hunk* gene in ES cells

E14Tg2a mouse ES cells were used to target *Hunk* allele as described in our previous publications [17]. Briefly, ES cells were transfected with NotI-linearised targeting plasmid by electroporation and clones were selected with G418 positive and gancyclovir negative selections. Clones were screened by PCR on genomic DNA template with primers MK1S 5′-tgagttgagggcttggtgttctttg-3′ located upstream of the “short arm” and neoB 5′-aagaacgagatcagcagcc-3′ located inside neomycin phosphotransferase expression sequence. Clones with homologous recombination of the “short arm” were identified by amplification of 1.1 kb fragment (Figure 1D). Homologous recombination of the “long arm” was analyzed by Southern blot analysis of genomic DNA digested with BamHI with P32-labeled probe “L”. While digestion of the wild type allele results in 10.3 kb fragment detected by hybridization, homologous recombination of the “long arm” lead to the appearance of an additional 9.3 kb fragment (Figure 1D).

### Generation of mice with targeted Hunk gene

Successfully targeted ES cell clones with normal chromosome complement were used for generating mouse chimeras by blastocyst (C57Bl/6 J) injection. The germ-line transfer was assessed by breeding male mouse chimeras with C57Bl/6 female mice. Presence of *Hunk +* and *Hunk-* alleles was confirmed by PCR with primers MKK (5′-tagtctggttggcatcaccg-3′), MK1A (5′-cagaatccagctagacctaacagtg-3′) and neoB on templates of genomic DNA isolated from mouse tail biopsies. Amplification with primers MK1A and MKK on *Hunk +* allele resulted in 383 bp amplification product while PCR with neoB and MKK primers on *Hunk* allele resulted in amplification of a 476 bp fragment (Figure 1D). PCR reaction contained 1 mM MgCl2 in Taq DNA polymerase reaction buffer, 0.2 mM dNTP, 2 U of Taq DNA polymerase (Fermentas), primers MKK, MK1A, neoB and genomic DNA template in the final volume of 25 μl. 35 cycles of 15 sec at 94°C, 30 sec at 63°C, and 60 sec at 72°C were carried out on DNA EngineDyad amplifier (BioRad).

### Mice and sample preparation

This study received ethical approval from Cardiff University’s Animal Welfare and Ethical Review Body (previously known as the ERP), and all animal procedures were conducted in accordance with UK Home Office regulations. AhCre + *Apc+/+* and AhCre + *Apcfl/fl* mice were generated and maintained on an outbred background as previously described [1]. Cre-recombinase activity was induced from the Ah-Cre transgene by three intra-peritoneal (IP) injections of 80 mg/kg β-naphthoflavone within 24 h. Mice were sacrificed at day 4. Cohorts containing the *Apc*^*Min*^ allele were sacrificed when animals displayed symptoms of intestinal disease, including weight loss, rectal bleeding and criteria of anaemia (as assessed by pale feet). Tissues were harvested, fixed and processed according to standard protocols as previously described [1].

### BrdU labelling

To achieve BrdU labelling for proliferation and migration studies, mice were administered with 250 μl BrdU (Amersham) via an IP injection either 2 hrs or 24 hrs prior to culling (n = 3 in all cases). Immunohistochemical (IHC) staining for BrdU was performed using an anti-BrdU antibody (BD biosciences 1:500). BrdU-positive cell position and number were scored. Kolmogorov–Smirnov test proved a significant difference between the distributions of BrdU-positive epithelial cells in crypts, 24 hr post BrdU administration.

### RT-PCR analysis

Total RNA extraction and first-strand cDNA synthesis were carried out as described previously [18]. For analysis of *Hunk* expression in mouse tissues one μl of cDNA was used as a template for PCR amplification with primers 5′-agatccagcagatgatccgac-3′ and 5′-tagcgctcaagtttcttgttcaa-3′ and Platinum AccuPrime DNA polymerase (Invitrogen). 35 cycles of 15 sec at 95°C and 90 sec at 68°C were carried out on DNA Engine Dyad amplifier (BioRad). qPCR was performed using Applied Biosystems TaqMan Universal PCR mix and Steponeplus machine. The 2 − ΔΔCT method [19] was used to calculate relative fold change in expression levels, with β-Actin expression being used as the housekeeping gene, which we can confirm amplified with an equivalent efficiency to the test primers. The mean ΔCT values for the experimental groups were compared to the mean ΔCT values for the control group, in order to provide the relative fold change. Thus, figures representing relative fold change do not possess error bars, although statistical significance between the ΔCT values was tested using the Mann–Whitney *U* test and deemed significant when p < 0.05. Primers used were: *Hunk* 5′atcacacagctccagagtacca3′ and 5′ggttggtgtggctctagtttct3′, β*-actin 5′*caccacaccttctacaatgagc3′ and 5′gtacgaccagaggcatacagg3′, *Axin2* 5*′*gcagctcagcaaaaagggaaat3′ and 5′tacatggggagcactgtctcgt3′, *Wif1* 5*′*aacaagtgccagtgtcgagagg3′ and 5′gcctttttaagtgaaggcgtgtg3′.

### Affymetrix microarray analysis

Normal colonic and paired polyp tissue was collected from symptomatic *Apc*^*Min*^ mice. Biotinylated target cRNA was generated from these tissues as previously described [1,20]. Affymetrix MOE430_2 gene arrays were run at the CRUK facility at the Paterson Institute for Cancer Research, and the data has been deposited in NCBI’s Gene Expression Omnibus and are accessible through GEO Series accession number GSE65461 (http://www.ncbi.nlm.nih.gov/geo/query/acc.cgi?acc=GSE65461). Arrays from AhCre + *Apc+/+* and AhCre + *Apcfl/fl* mice have previously been published for intestinal tissue [1] and liver tissues [20].

## Results

### Wnt signalling activation results in up-regulated *Hunk* expression

**Figure 2:**
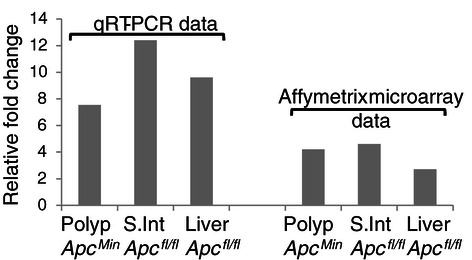
**A Relative fold change in the levels of*Hunk*expression compared to the appropriate normal tissue assessed using qRT-PCR analysis and Affymetrix micro-array (Intestinal array[**1**],liver array[**20**]).** In all cases Mann–Whitney *U* test reveals a significant difference between ΔCT values (p < 0.05). Small intestine (S. Int) and liver samples were collected Day 4 post Ah-Cre induction. Normal colonic tissue and adjacent adenoma tissue (polyp) were taken from *Apc*^*Min*^ animals.

Apc is a known key regulator of Wnt signalling, and critically important in regulating normal intestinal homeostasis. Conditional deletion of *Apc* within the mouse intestine using an Ah-Cre recombinase to drive recombination of LoxP flanked *Apc* alleles, has previously been shown to result in acute activation of Wnt signalling and many hallmarks of neoplasia, including increased proliferation and apoptosis and loss of differentiation and migration [1]. Affymetrix microarray analysis indicates an acute transcriptional activation of *Hunk* following the loss of *Apc* in the intestine and liver and in colonic adenomas from the *Apc*^*Min*^ mouse (Figure 2). qRT-PCR analysis confirms the transcriptional activation of *Hunk* in these settings (Figure 1), indicating that *Hunk* transcription is coincident with Wnt signalling activation and tumour formation. Indeed, a Tcf/LEF consensus binding site can be found within the promoter region of *Hunk*, and significant up-regulation of *HUNK* has been shown to occur in human colorectal cancer cell lines [12].

### Hunk-kinase deficiency results in increased intestinal cell proliferation

To assist in our quest to investigate the importance of Hunk-kinase in intestinal tumourigenesis, we generated a novel mouse line carrying a Hunk-kinase deficient allele. To do this exon 4 of the mouse *Hunk* gene was targeted. A protein fragment encoded by this exon (amino acids 204–249) contains a part of subdomain VII (starting from conserved Asp204 which is important for γ-phosphate of MgATP orientation), the entire subdomain VIII, which is critical for substrate recognition, and a portion of subdomain IX of the Hunk protein kinase catalytic domain. Therefore, deletion of exon 4 results in the production of a catalytically inactive Hunk protein. Moreover, deletion of the exon 4 results in a shift of the open reading frame in the transcript after joining exons 3 and 5. As a result, the translated protein would consist of only 203 amino acids of the Hunk polypeptide with translation terminating 2 codons downstream of the codon encoding Ile203 (Figure 1А and B). Following successful targeting in ES cells (Figure 1D, F) and the production of chimeric animals after ES cell injection into blastocysts, successful germ line transmission of the targeted allele was confirmed by PCR (Figure 1G). Transgenic mice were further back-crossed for 6 generation to obtain a mouse line on a pure C57Bl/6 J genetic background.

Due to the lack of suitable antibodies for the detection of endogenous Hunk protein in mouse tissue, RT-PCR was used to analyze *Hunk* transcripts in wild type, *Hunk-* heterozygous and homozygous mice (Figure 1 H,I). The *Hunk+/+* yielded the expected 826 bp PCR fragment corresponding to the wild type *Hunk* allele, while it was completely absent in samples of *Hunk−/−* animals (Figure 1I). However, along with a 690 bp PCR fragment corresponding to mRNA lacking exon 4, an additional prominent amplification product, a 562 bp fragment, was detected in *Hunk−/−* animals (Figure 1I). Cloning and sequencing revealed that this fragment represents a *Hunk* transcript lacking not only targeted exon 4 but also exon 5 sequences. Importantly, deletion of both exons 4 and 5 (Figure 1H), while resulting in transcript encoding catalytically inactive protein due to deletion of catalytic domain portion, does not lead to a frame-shift and the translated protein should be identical to full-length Hunk except for the deletion of amino acids 204–291 (Figure 1C). In heterozygous *Hunk+/−* mice, the wild type allele transcript was substantially more abundant than both variants of the mutant allele transcript (Figure 1I).

**Figure 3:**
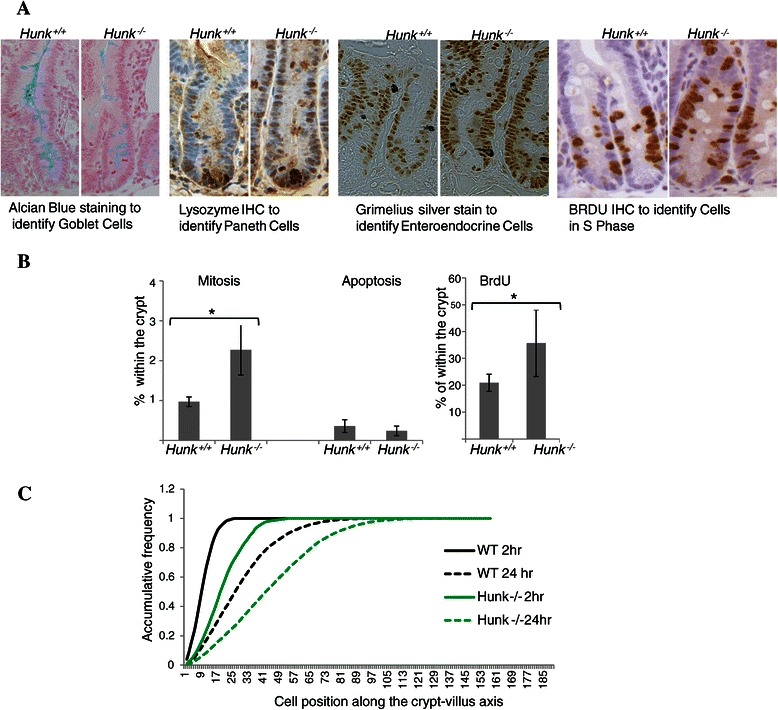
**Characterisation of the intestine following Hunk-kinase loss. A** Representative images showing no difference in Alcain Blue staining (Goblet cells) and Lysozyme IHC (Paneth cells) in the different genotypes, and an increase in BRDU incorporation 2 hours post administration. **B** Haematoxylin and eosin stained intestinal sections were used to score the percentage of Mitotic and Apoptotic bodies within intestinal crypts, while BrdU IHC stained sections were used to score BrdU incorporation 2 hours post administration. Bar charts show means ± SD determined by scoring at least 50 half crypts within 4 individuals from each cohort. * denotes p < 0.05 Mann–Whitney *U* test. **C** Accumulative frequency of BrdU positive cell position along the crypt-villus axis, 2 hours and 24 hours post administration. Significant differences between the genotypes were detected at both time points using Kolmogorov–Smirnov test, a test designed to examine probability distribution patterns.

Given our interests in the role of Hunk in intestinal tumourigenesis, a detailed examination of the phenotype in *Hunk−/−* intestine was performed. No differences were found in the representation of the different cell types of the intestine (assessed using alcian blue staining for goblet cells, lysozyme IHC for paneth cells and grimelius staining for enteroendocrine cells), suggesting Hunk-kinase activity is not involved in lineage specification in the intestine (Figure 3A). However, although the gross morphology remained unaltered, with the number of cells within the crypt remaining the same, *Hunk−/−* intestine displayed a significant increase in crypt cell proliferation within the small intestines (scored using BrdU incorporation and histological examination of intestinal crypts, Figure 3B). This was not accompanied by any alteration in the rates of apoptosis (Figure 3B), although migration rates along the crypt-villus axis were significantly perturbed; *Hunk−/−* intestinal cells display an increased rate of migration (Figure 3C). Consequently, these data demonstrate that within a normal intestinal setting, loss of kinase active Hunk was sufficient to induce alterations in the normal intestinal kinetics, but this did not alter normal intestinal physiology.

### Hunk-kinase deficiency alters tumour initiation rates but not survival in *Apc*^*Min*^ mice

**Figure 4:**
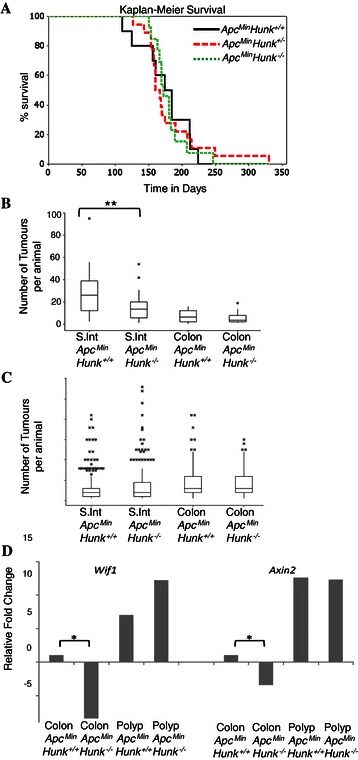
**Survival and tumour burden analysis. A** Kaplan-Meier survival curve of aged cohorts of *Apc*^*Min*^*Hunk*^*+/+*^ (n = 23), *Apc*^*Min*^*Hunk*^*+/−*^ (n = 27) and *Apc*^*Min*^*Hunk*^*−/−*^ (n = 23) mice, demonstrating that no significant differences in survival between the cohorts. **B** Box plot displaying the total number of tumours found at death in the aged cohorts of *Apc*^*Min*^*Hunk*^+/+^ and *Apc*^*Min*^*Hunk*^−/−^ mice. The box encompasses the first quartile (at bottom) to the third quartile (at top) of the data set and the horizontal boxed line represents the median. ** p < 0.01 Mann–Whitney *U* test. **C** Box plot displaying the size of tumours found at death in the aged cohorts of *Apc*^*Min*^*Hunk*^+/+^ and *Apc*^*Min*^*Hunk*^−/−^ mice. **D** qRT-PCR analysis showing relative expression levels of *Axin2* and *Wif1* in normal colonic tissue (colon) and adjacent adenoma tissue (polyp) taken from 4 aged matched animals from the different genotypes. * p < 0.05 Mann–Whitney *U* test between ΔCT values.

In order to address the importance of Hunk-kinase in Wnt driven intestinal tumourigenesis, the Hunk-kinase deficient mice were inter-crossed with the established *Apc*^*Min*^ mouse model of intestinal cancer. Cohorts of *Apc*^*Min*^*Hunk*^+/+^, *Apc*^*Min*^*Hunk*^+/−^ and *Apc*^*Min*^*Hunk*^−/−^ littermates were generated and aged and monitored until the animals displayed overt symptoms of intestinal disease, at which stage they were culled using the appropriate schedule 1 method, and tissues were harvested. Kaplan-Meier survival analysis demonstrated that loss of kinase active Hunk does not alter the survival of *Apc*^*Min*^ mice (Figure 4A). However, macroscopic scoring of tumours at dissection showed significantly fewer (p = 0.025 Mann–Whitney), yet larger tumours in the *Apc*^*Min*^*Hunk*^−/−^ cohort (mean number 21.4per animal +/− 2.7SEM, mean size 7.1 mm2 +/− 0.3SEM) compared to the *Apc*^*Min*^*Hunk*^+/+^ cohort (mean number 36.2 per animal +/− 4.8SEM, mean size 5.7 mm2 +/− 0.3SEM). Furthermore, this difference was restricted to the small intestine (Figure 4B). Detailed microscopic analysis of these tumours did not reveal any differences in the stage, types or characteristics of the tumours occurring in the different cohorts. Neither the proliferation rates nor apoptosis rates within tumours differed with Hunk-kinase status (Additional file 1: Figure S1). Furthermore, qRT-PCR analysis for Wnt target genes (including *cMyc, Ascl2, Axin2, CD44, CD1, Sox17, Wif1* and *Tiam1*) did not identify any significant differences between the colonic tumours isolated from both cohorts of mice. However, it is pertinent to note that both *Wif1* and *Axin2* were significantly down-regulated within the normal colonic *Apc*^*Min*^*Hunk*^−/−^ tissue compared to normal colon samples from *Apc*^*Min*^*Hunk*^+/+^ mice (Figure 4D). Thus, kinase active Hunk positively contributes toward the normal expression of two negative regulators of canonical Wnt signalling within normal intestinal tissue, but loss of Hunk-kinase and the subsequent down regulation of *Axin2* and *Wif1* does not confer a generic mis-regulation of Wnt signalling. A precise molecular characterisation following the loss of Hunk-kinase is required to ascertain the exact mechanism by which this kinase influences gene transcription and cell proliferation. Overall, in relation to intestinal tumourigenesis our results show that Hunk-kinase activity does impact on intestinal tumour initiation within the small intestine, but this is not sufficient to alter the overall tumour burden or survival in the *Apc*^*Min*^ mouse model of intestinal cancer.

## Discussion

The exact role of the SNF1-related serine/threonine kinase Hunk (Mak-V) remains unclear. Here we have generated a novel kinase-deficient Hunk allele, and produced homozygous Hunk-kinase deficient mice, *Hunk−/−*. Two previous studies have shown Hunk to be a negative regulator of proliferation within normal epithelial cells [9,10] and our findings support this notion. We have shown that loss of Hunk-kinase activity within the normal intestinal setting results in an increase in proliferation within epithelial cells. This is accompanied by an increase in cell migration rates, thereby maintaining normal physiology despite altered kinetics.

An increasing body of evidence links the function of Hunk with cancer initiation, progression and metastasis [3,8,11-13], although there remains uncertainty regarding the precise involvement of *Hunk* in tumourigenesis. To date, most studies have analysed the role of Hunk in mammary tumourigenesis, largely due to the known role of this protein in mammary gland development [4]. However, our microarray findings implicated *Hunk* in intestinal tumourigenesis, a role we wished to elucidate further. We have shown that *Hunk* expression becomes significantly up-regulated from the earliest stages of tumour initiation following *Apc* loss, indicating this gene is probably a Wnt signalling target gene. Indeed a Tcf/LEF binding motif can be found within the promoter region of *Hunk.* We appreciate this evidence is circumstantial, and a more detailed interrogation is required to confirm *Hunk* as a Wnt target gene.

Studies using Xenopus embryos have shown that Hunk has the ability to modulate Wnt signalling. We used qRT-PCR analysis to examine this in the intestine and demonstrated a significant reduction in expression levels of two negative regulators of Wnt signalling, *Wif1* and *Axin2*, accompanying the loss of kinase active Hunk. However, this did not translate to a generic de-regulation of Wnt signalling. Interestingly, contrary to a recent publication by Yeh *et al.* [14] who described Hunk as a negative regulator of cMyc expression, we did not observe any significant alteration in the levels of cMyc transcription accompanying Hunk-kinase loss. Discrepancies in the examined tissues and experimental setup might account for these differences. In an attempt to explain the mis-regulation of both *Wif1* and *Axin2* which can be regulated by components of the BMP/TGFβpathway [21,22], qRT-PCR analysis of components and targets of this pathway was performed. However once more, a generic mis-regulation of this pathway was not confirmed by qRT-PCR analysis. A more detailed genomic wide study would be required to confidently identify the mechanism through which Hunk-kinase is able to negatively regulate proliferation in the intestine.

Intercrossing *Hunk*−/− mice with *Apc*^*Min*^ mice allowed us to determine the role of Hunk-kinase in Wnt signalling driven intestinal tumourigenesis. We have shown a significant reduction in the tumour initiation rate within the small intestine in *Apc*^*Min*^*Hunk*^−/−^ mice, but this does not impact on overall survival due to an accompanying increase in the size of those tumours that do form. It is possible that the reduced tumour initiation rate is associated with the increased cell turnover rate along the crypt-villus axis (increased proliferation and migration) seen following the loss of Hunk-kinase, although the exact mechanisms for this have not been elucidated. Further studies would be required to determine the significance of these subtle changes associated with the lack of kinase active Hunk.

Overall, our data confirm Hunk-kinase as a negative regulator of normal epithelia proliferation, and demonstrate that in the classical *Apc*^*Min*^ mouse model of intestinal tumourigenes, Hunk-kinase activity significantly impacts on tumour initiation rates during the early stages in tumourigenesis.

## Conclusions

Here we describe the production of a new Hunk-kinase deficient mouse model and use it to examine the importance of this kinase during the early stages of intestinal tumourigenesis. We show that despite not affecting overall survival of the *Apc*^*Min*^ mice, Hunk-kinase is a negative regulator of normal intestinal proliferation, and impacts significantly on small intestinal tumour initiation rates.

## Availability of supporting data

The Affymetrix array data has been deposited in NCBI’s Gene Expression Omnibus and are accessible through GEO Series accession number GSE65461 (http://www.ncbi.nlm.nih.gov/geo/query/acc.cgi?acc=GSE65461).

## Additional file

Additional file 1: Figure S1. Mitosis and apoptosis levels scored from H + E stained sections on intestinal adenomas. Bar charts show means SD of values obtained from at least 6 tumours from three individuals within each cohort.

## Abbreviations

APC: Adenomatosis polyposis coli
ES: Embryonic stem cell
HUNK: Hormonally upregulated Neu-associated kinase
IHC: Immunohistochemistry
IP: Intra-peritoneal
MDCT: Mouse distal convoluted tubule
S. Int: Small intestine
