# Supplementary figures and images for: Hunk/Mak-v is a negative regulator of intestinal cell proliferation
Source: BMC Cancer. 2015 Mar 8;15:110. doi: 10.1186/s12885-015-1087-2 (PMC4367870; doi:10.1186/s12885-015-1087-2)

## Mitosis in adenoma

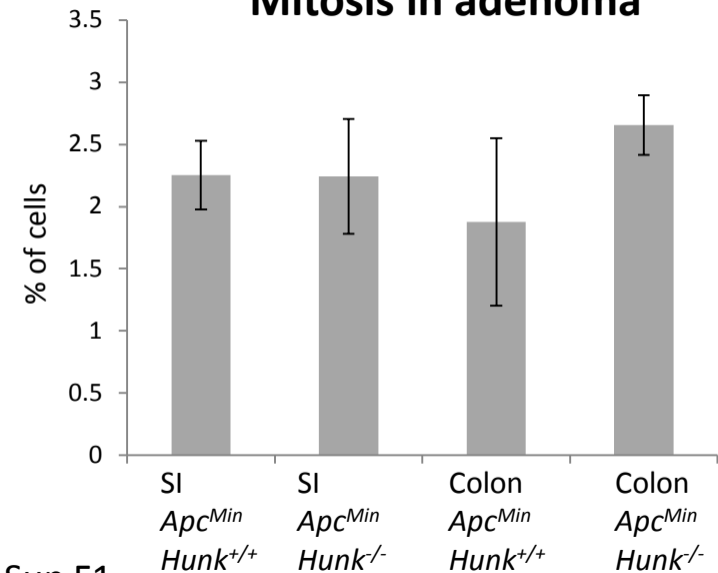

## Apoptosis in adenoma

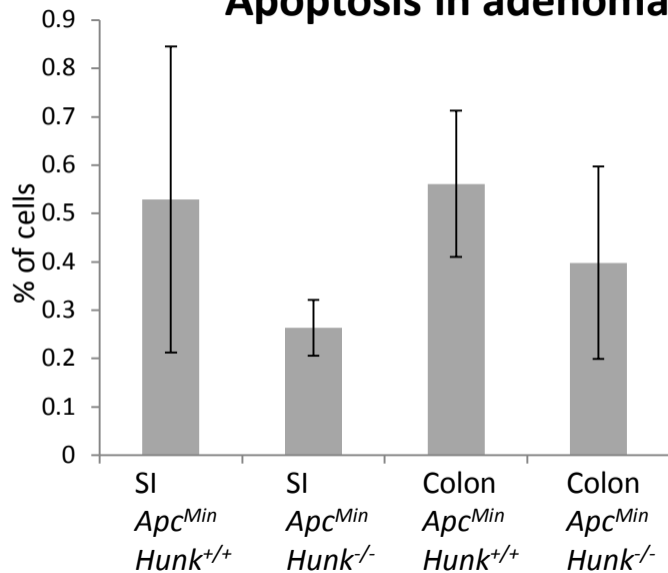

Supplement: Additional file 1: Figure S1. — Mitosis and apoptosis levels scored from H + E stained sections on intestinal adenomas. Bar charts show means SD of values obtained from at least 6 tumours from three individuals within each cohort. [file 12885_2015_1087_MOESM1_ESM.pdf]
